# Mammary mucinous cystadenocarcinoma with long-term follow-up: molecular information and literature review

**DOI:** 10.1186/s13000-023-01302-2

**Published:** 2023-02-04

**Authors:** Ting Lei, Yong Qiang Shi, Tong Bing Chen

**Affiliations:** Department of Pathology, The Third Affiliated Hospital of Soochow University, Ju Qian Street 185, Changzhou, 213003 Jiangsu China

**Keywords:** Mucinous cystadenocarcinoma, Breast, Genomic features, Follow-up

## Abstract

**Background:**

Mucinous cystadenocarcinoma (MCA) is a very rare form of breast cancer that was first described in 1998. Only 33 cases of primary MCA, including our present case, have been reported thus far. As a consequence, its molecular features, prognosis and treatment regimen are poorly known. Here, we describe a less common presentation of MCA, detail its molecular features, discuss the major differential diagnosis, and provide a brief review of the literature.

**Case presentation:**

A 59-year-old woman presented with a breast lump in which mammography showed a well-defined nodule. Core needle biopsy (CNB) revealed several lesions lined by tall columnar cells with stratification and abundant mucinous secretion; excision was recommended for final diagnosis. The resected specimens showed cavities of different sizes without surrounding myoepithelial cells. The cavities were rich in mucus, and the nuclei were located at the base of the cells, containing intracellular mucus. Immunohistochemical analysis revealed that it was triple-negative breast cancer (TNBC). Next-generation sequencing (NGS) revealed pathogenic mutations in the *PIK3CA*, *KRAS*, *MAP2K4*, *RB1*, *KDR*, *PKHD1*, *TERT*, and *TP53* genes. A diagnosis of MCA was rendered. The patient has been followed up for 108 months to date and showed no signs of recurrence or metastasis.

**Conclusion:**

Our study presents the gene profile of an MCA case with no recurrence or metastatic tendency after 108 months of follow-up, and a review of the literature helps us better understand the clinical, pathologic, and molecular features of this tumor.

## Introduction

Mucinous cystadenocarcinoma (MCA) is an exceptionally rare variant of primary breast cancer that was first described by Tavassoli et al. in 1998, with approximately 30 cases reported in the English literature [1, 2]. MCAs are characterized by high columnal cells that are rich in intracellular mucin, and the lumen contains a large amount of extracellular mucin. The diagnostic process is challenging in some cases due to overlapping histological characteristics with other lesions. Accurate morphological recognition, understanding the immunohistochemical and molecular features of such diseases, and avoiding improper management are essential.

In the current limited studies, MCAs typically occurred in postmenopausal females with a median age of 61 years [1]. Patients were followed up from 3 to 96 months and had a relatively good prognosis without distant metastasis [2–29]. The entity is usually triple-negative breast cancer (TNBC), which is negative for estrogen receptor (ER), progestogen receptor (PR) and human epidermal growth factor receptor 2 (HER2) expression [2–29]. Due to its rarity, the pathogenesis and prognosis of this disease remain poorly characterized. Additionally, a standard treatment regimen is still lacking.

Herein, we report a case of MCA in a 59-year-old woman without evidence of recurrence or metastasis at 108 months after surgery. Genomic profiling was performed, providing evidence for a better understanding of this rare tumor.

## Case presentation

**Fig. 1:**
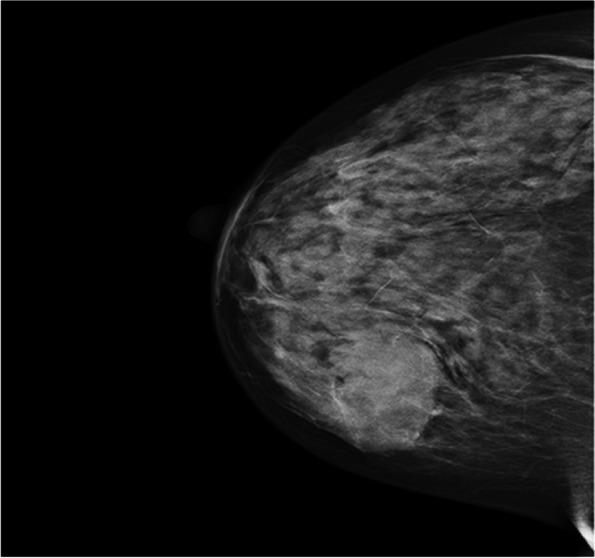
Mammography image of the lesion, revealing a well-defined nodule within the breast

A 59-year-old postmenopausal female presented herself to our hospital with a mass on her right breast for 2 weeks. The patient had no history of hormonal treatment or family history of cancer. Clinical examination confirmed a nodule in the right breast, situated at 5 o’clock. Mammography revealed a spherical, well-defined nodule of 3.2*3*2.3 cm (Fig. 1).

**Fig. 2:**
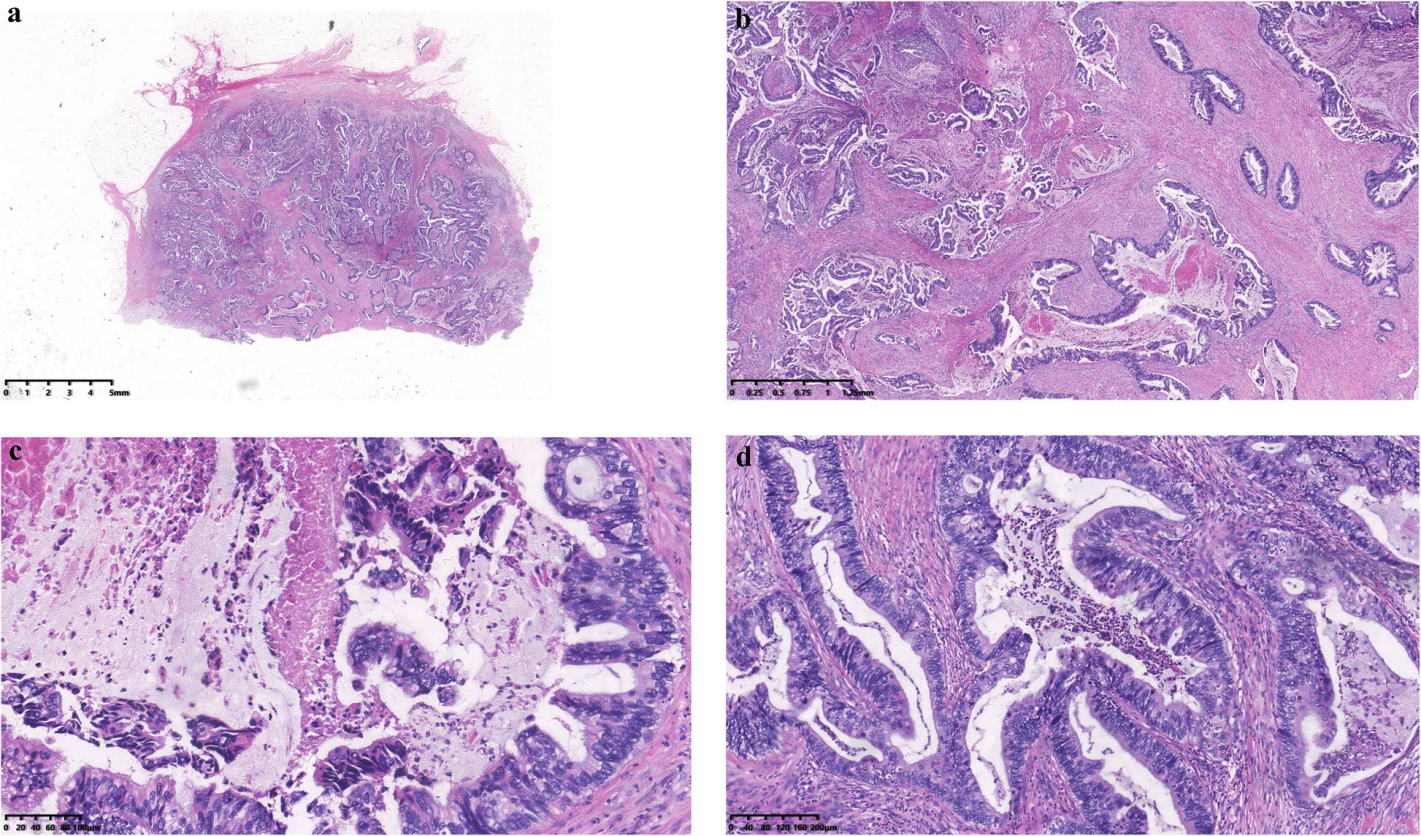
**a** The low-power view illustrates that the surgical specimen was a well-circumscribed tumor. **b**, **c**, **d** The tumor consisted of mucus-filled cavities of varying sizes lined with columnar cells (**b**, low-power). Tall columnar cells were rich in mucous and had nuclei at the base of the cell. Some areas were stratified, protruding into the lumen and even forming branched papillary structures. Nests or papillary cell masses floated in the intracavity mucous lake accompanied by inflammatory cell infiltration (**c**, **d** high-power)

**Fig. 3:**
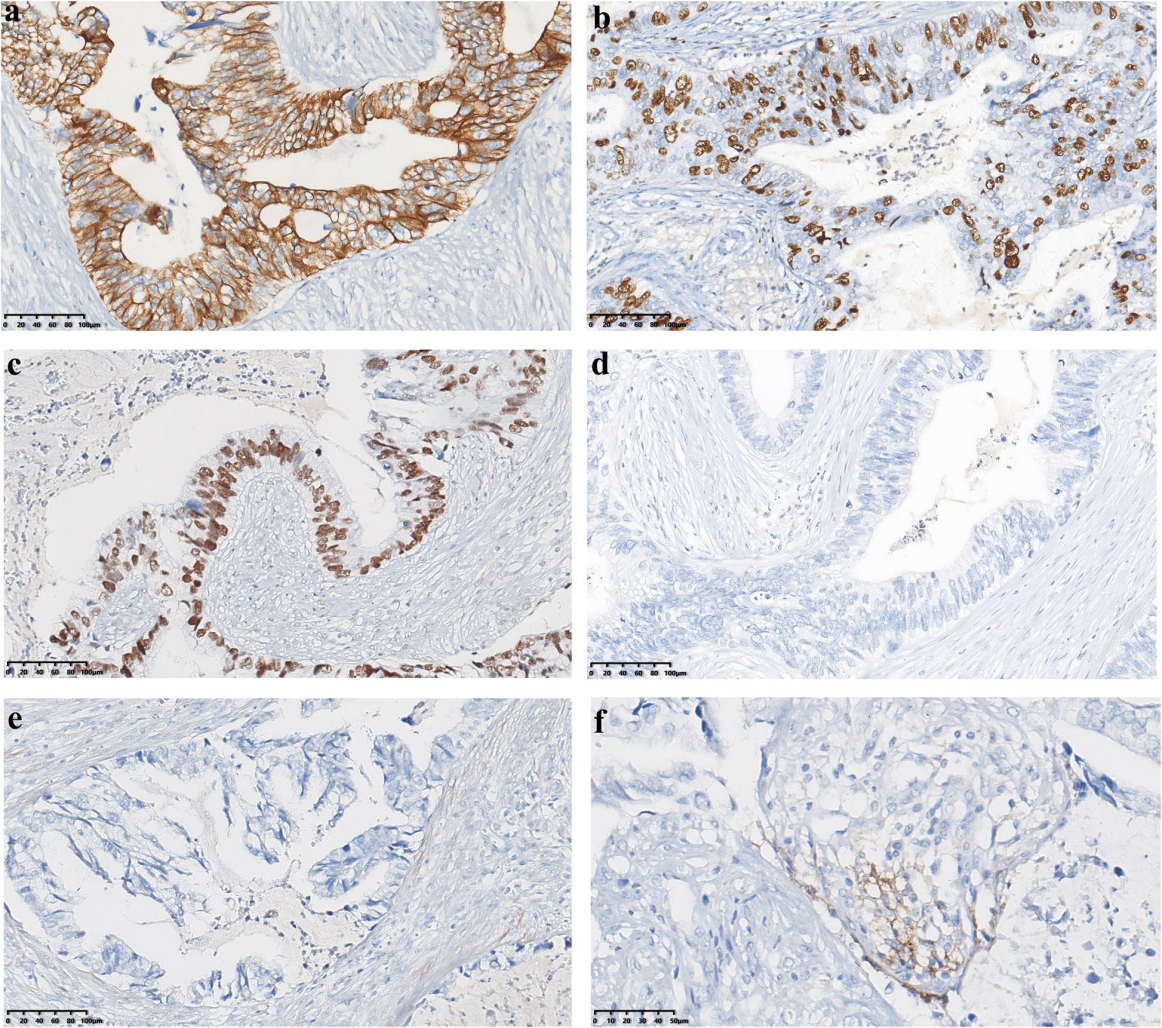
Immunohistochemical features of the lesion. **a** Cytokeratin 7 was strongly and diffusely positive for neoplastic cells. **b** The Ki-67 index of the tumor cells. **c** Overexpression of p53 protein. **d** Myoepithelial markers were absent (p63). **e** Myoepithelial markers were absent (calponin). **f** Focal positive expression of PD-L1 in immune cells within the tumor

Core needle biopsy (CNB) revealed multiple lesions lined with layered columnar cells and abundant mucous secretion, and the diagnosis of invasive breast cancer with abundant mucous secretion was made. Then, a right lumpectomy along with ipsilateral axillary lymph node dissection was performed. Under macroscopic observation, the tumor was a well-circumscribed mass 3*3*2 cm in size. The cross-section was grayish-white with a moderate myxoid appearance. Microscopically, the tumor consisted of mucus-filled cavities of varying sizes lined with columnar cells (Fig. 2a, b). Tall columnar cells were rich in mucous and had nuclei at the base of the cell. Cells in some areas appeared stratified, protruding into the lumen and even forming branched papillary structures. Nests or papillary cell masses floated in the intracavity mucous lake accompanied by necrosis and inflammatory cell infiltration (Fig. 2c, d). Microscopically, no distinct myoepithelial layer was observed, and subsequent immunohistochemical results also confirmed the absence of myoepithelium (Fig. 3d, e). The cells had mild atypia, and mitotic figures were rare. No common ductal carcinoma in situ (DCIS) existed, and ipsilateral axillary lymph nodes showed no metastasis. The Nottingham grade was 1 (tubule formation = 3, nuclear pleomorphism = 1, and mitotic count = 1), and the pathological stage was T2N0Mx.

Based on these morphological features, a wide range of differential diagnoses included metastatic tumors from the ovaries or pancreas, mucinous carcinoma, mucoceloid lesions, encapsulated papillary carcinoma (EPC) and invasive papillary carcinoma. A broad immunohistochemical panel was performed to narrow the differential diagnosis. The neoplastic cells showed diffuse immunoreactivity for cytokeratin 7 (CK7) (Fig. 3a) and a high Ki-67 index of up to 40% (Fig. 3b). There was no immunoreactivity for ER, PR, HER2, cytokeratin 20 (CK20), CA19-9, CDX-2, Villin, PAX8, GATA3, SOX10, GCDFP-15, mammaglobin, p63 or calponin. Positive immunoreactivity for CK7, negative immunoreactivity for CK20, CA19-9, CDX-2, and Villin, and metastasis from the ovary, pancreas or intestine were excluded. Positron emission tomography (PET)/computed tomography (CT) was performed on the patient, and no other lesions were found, confirming nonmetastatic lesions. A triple-negative immunophenotype and a relatively high Ki-67 index ruled out mucinous carcinoma and EPC, which typically express ER and PR. Mucoceloid lesions of the breast are benign lesions in which myoepithelium is present around the lumen. The absence of myoepithelium also ruled out this diagnosis (Fig. 3d, e). Invasive papillary carcinoma is composed of mildly dilated ducts and microcysts containing a papillary formation without intracellular and extracellular mucus. These cases are usually non-triple-negative phenotypes. Eventually, we favored the diagnosis of MCA based on the morphological and immunohistochemical findings.

Furthermore, 425 genes were sequenced using formalin-fixed and paraffin-embedded (FFPE) tissues and next-generation sequencing (NGS) technology. Recurrent mutations in *PIK3CA*, *KRAS*, *MAP2K4*, *RB1*, *KDR*, *PKHD1*, *TERT*, and *TP53* were identified and are summarized in Table 1. The tumor mutation burden (TMB) was 9.27, and microsatellite instability high (MSI-H) was not detected. P53, RB1 and PD-L1 protein were stained according to the sequencing results. Immunohistochemistry confirmed the overexpression of p53 protein (Fig. 3c) and loss of RB1 protein expression. PD-L1 (sp142) was focally positive in immune cells, and the positive rate was approximately 7% (Fig. 3f). The patient received 6 cycles of adjuvant chemotherapy and was followed up for 108 months, with no signs of recurrence or metastasis.

**Table 1:** Summary of the genetic profile identified in a case of primary mucinous cystadenocarcinoma of the breast and review of the literature

Cases	gene	Type of mutation	Mutation site	Allele frequency
Present case	PIK3CA	Missense mutation	c.3140A	23.9%
	KRAS	Missense mutation	c.35G	55.5%
	MAP2K4	Frameshift mutation	c.257_258del	30.5%
	RB1	nonsense mutation	c.277C	55.2%
	KDR	Missense mutation	c.521G	14.1%
	PKHD1	Missense mutation	c.6453G	30.9%
	TERT	Missense mutation	c.1006G	15.5%
	TP53	Missense mutation	c.476C	44.0%
Lin’s case [18]	BAP1	Frameshift deletion	c.362delG	6.5%
	RB1	Frameshift deletion	c.2518delG	9.4%
	TP53	Missense mutation	c.G329C	7.7%

## Discussion

Table 2 summarizes the features of MCAs previously described and our present case [2–29]. To date, only 33 cases, including our present case, have been reported in the English literature [2–29]. Overall, MCA predominantly affected perimenopausal or postmenopausal women aged 41 to 96 years [2–29]. The tumor size ranged from 0.8 cm to 19 cm, and there were two cases with multiple nodes [2–29]. Metastatic lymph nodes were seen in 5 cases, with no more than 3 lymph nodes involved [2, 4, 13, 20, 21].

**Table 2:** Comparison of the clinico-pathologic features of our case with other cases reported

Number	Cases	Patient profile	Tumor size (cm)	pN	ER	PR	HER2	Ki-67	CK7	CK20	Associated Findings	Surgery; CT/RT/HT	Follow-up (FU)
1	Rosen PP et al.[22]	79 years; F	6.0	NA	**_**	**_**	NA	NA	NA	NA	NA	M, LND	9 years +
2	Tavasolli et al.[2]	54 years; F	19.0	N2	**_**	**_**	NA	40%	+	**_**	none	M, LND	24 months ANED
3		67 years; F	2.3	N0	**_**	**_**	NA	30%	+	**_**	DCIS	M, LND	22 months ANED
4		49 years; F	8.5	N0	**_**	**_**	NA	70%	+	**_**	DCIS	M, LND, CT + RT	11 months ANED
5		61 years; F	0.8	N0	**_**	**_**	NA	50%	+	**_**	none	Lumpectomy, LND	NA
6	Domoto H, et al.[6]	74 years; F	10.0	N0	**_**	**_**	**_**	22%	+	**_**	NA	M, LND,	2 years, ANED
7	Naoko H et al.[20]	96 years; F	2.0	N1	**_**	**_**	_	35%	NA	NA	none	Lumpectomy, LND	46 months +
8	Chen et al.[27]	65 years; F	3.0	N0	**_**	**_**	_	NA	+	**_**	IDC, DCIS	M, LND, CT	8 months, ANED
9	Coyne JD et al.[3]	51 years; F	4.0	N0	**_**	**_**	NA	NA	+	**_**	NA	Lumpectomy,	NA
10	Lee et al.[15]	55 years; F	2.5	N0	**_**	**_**	_	10%	+	**_**	IDC, DCIS	M, LND	6 months, ANED
11	Rakici et al.[24]	52 years; F	10.0	N0	+	**_**	_	NA	**_**	**_**	ADH	M, LND, HT	24 months, ANED
12	Lin et al.[5]	61 years; F	3.0	N0	**_**	**_**	_	NA	NA	**_**	none	M, LND	6 months, ANED
13	Petersson et al.[23]	73 years; F	4.5	N0	**_**	**_**	2 + (FISH +)	NA	+	**_**	DCIS	M, LND	NA
14	Sentani et al.[25]	65 years; F	3.0	N0	**_**	**_**	NA	NA	+	**_**	DCIS	Lumpectomy, LND	6 months, ANED
15	Witherspoon et al.[28]	91 years; F	7.5	N0	**_**	**_**	_	40%	+	**_**	IDC, DCIS	Lumpectomy, LND, RT	14 months, ANED
16	Deng et al.[4]	41 years; F	7.0, 5.0, 2.5	N3	**_**	**_**	_	50%	+	**_**	DCIS	M, LND	24 months, ANED
17	Li et al.[16]	52 years; F	6.5	N0	**_**	**_**	_	10%	+	**_**	none	NA	NA
18	Kim et al.[12]	59 years; F	0.9	N0	**_**	**_**	2 + (FISH -)	5%	+	**_**	IDC, DCIS	Lumpectomy, LND, CT	3 months, ANED
19	Koufopoulos et al.[13]	63 years; F	1.6	N2	**_**	**_**	_	NA	+	**_**	none	M, LND	48 months, ANED
20	Lin et al.[17]	62 years; F	5.6	N0	**_**	**_**	_	NA	+	**_**	none	M, LND	5 months, ANED
21	Kucukzeybek et al.[14]	55 years; F	2.0	N0	**_**	**_**	2 + (FISH +)	30%	+	**_**	DCIS	M, LND, CT, RT, trastuzumab	NA
22	Seong et al.[26]	59 years; F	2.0	NA	**_**	**_**	3 +	NA	NA	NA	NA	NA	NA
23		50 years; F	2.2	NA	**_**	**_**	_	NA	NA	NA	NA	NA	NA
24	Nayak et al.[21]	68 years; F	6.2	N0	**_**	**_**	_	NA	+	**_**	DCIS	M, LND	3 months, ANED
25		51 years; F;	2.0	N1	**_**	**_**	_	NA	+	**_**	DCIS	M, LND	8 years, recurrence
26	Kaur et al.[11]	45 years; F	12.0	NA	**_**	**_**	_	NA	+	**_**	none	M	NA
27	Wang et al.[29]	66 years; F	2.5	N0	**_**	**_**	_	60%	+	**_**	DCIS	NA	10 months, ANED
28	Sun et al.[19]	56 years; F	1.0, 1.5, 1.2, 2.0	N0	+	** + **	1 +	3%-5%	**_**	**_**	lobular lesions	M, LND, HT	3 years, ANED
29	Lin et al.[18]	72 years; F	0.9	N0	**_**	**_**	_	30%	+	**_**	none	M, LND, RT	16 months, ANED
30	Kamrani et al.[9]	69 years; F	2.0	N0	**_**	**_**	_	NA	NA	NA	DCIS	M	NA
31	Ekta et al.[8]	45 years; F	4.3	N0	**_**	**_**	_	45%-50%	+	+	DCIS	M, LND, CT	NA
32	Kaur et al.[10]	65 years; F	18.0	N0	**_**	**_**	3 +	90%	+	focal +	none	M	6 months, ANED
33	Present case	59 years; F;	3.0	N0	**_**	**_**	_	40%	+	**_**	NA	M, LND, CT	108 months, ANED

*Abbreviation*: *F* Female, *NA* Not available, *CT* Chemotherapy, *RT* Radiotherapy, *HT* Hormonotherapy, *ANED* Alive with no evidence of disease, *M* Mastectomy, *LND* Lymph node dissection,  + Died of disease other than carcinoma, *PN* Pathology lymph node, *ER* Estrogen receptor, *PR* Progestogen receptor, *HER2* Human epidermal growth factor 2, *FISH* Fluorescence in situ hybridization, *IDC* Invasive ductal carcinoma, *DCIS* Ductal carcinoma in situ

The morphological spectrum of MCA ranges from pure MCA and MCA with DCIS to MCA with both DCIS and invasive ductal carcinoma (IDC) [2–29]. The diagnosis of MCA without DCIS is challenging because overlapping morphological features are not uncommon among the entities, suggesting the use of broad immunohistochemical biomarkers. The combination of clinical history, morphology, and immunohistochemistry is helpful to confirm the diagnosis. The immunohistochemical staining of our present case showed that only CK7 was positive. To exclude ovarian or pancreatic metastatic cancer, we added markers of ovarian and pancreatic origin, including CK20, CA19-9, CDX-2, Villin and PAX8, all of which were negative. In terms of the immunohistochemical phenotype, ovarian and pancreatic mucinous adenocarcinomas are usually CK7 + /CK20 + , while MCA of the breast is usually CK7 + and CK20– [24]. Therefore, a group of biomarkers is recommended for differential diagnosis, in which CK7 + /CK20- may assist in the diagnosis of the primary breast lesion, but a detailed clinical evaluation is required [24]. Additionally, various primary breast lesions are considered for the differential diagnosis of MCAs, including mucoceloid lesions, mucinous carcinoma, EPC and invasive papillary carcinoma [10, 13, 30–32]. The presence of mucinous cells, invasive growth behavior, and loss of myoepithelial expression are the first characteristics used to rule out mucinous cyst lesions [31]. The presence of intracellular and extracellular mucus and a triple-negative phenotype exclude the diagnosis of invasive papillary carcinoma [30, 33]. Additionally, the triple-negative phenotype and a relatively high Ki-67 index help to exclude mucinous carcinoma and EPC [10, 13, 30–32].

For the molecular subtype, two hormone receptor-positive, twenty-two triple-negative and four HER2 overexpression cases were among the known molecular phenotypes [2–29]. The Ki-67 index ranged from 3 to 99%, with most cases having a Ki-67 index higher than 30% [2–29]. Only one case recurred eight years after surgery, and no cases of metastasis have been reported [2–29]. One reason is that such tumors may be indolent tumors. However, unlike that in other low-grade indolent TNBCs, Ki-67 in most MCAs is relatively high. The other reason is that only three cases were followed up for more than five years, and the follow-up times of the remaining cases were approximately 1–2 years [2–29]. The biological behavior of the tumor was not fully demonstrated during the short follow-up period. MCAs may have a long-term risk of local recurrence, and whether they have metastatic potential requires more accumulated cases. Many clinicopathological parameters, including ER, PR, HER2, Ki-67, tumor size, tumor grade, lymph node status, and vascular invasion, have prognostic significance in breast cancer [34]. For TNBC, the prognostic value of parameters such as tumor grade, tumor size and lymph node status are still questionable; in contrast, high expression of Ki-67 and overexpression of p53 protein may contribute to poor prognosis in such tumors [35, 36]. Our case was a TNBC with high expression of the Ki-67 index and overexpression of p53 protein, suggesting that the patient had some adverse prognostic factors. The only reported recurrent case was a microinvasive breast cancer with the triple-negative phenotype and a small tumor size, but the expression of Ki-67 and p53 protein was unknown [21]. Therefore, more long-term follow-up cases are needed to verify the prognostic factors of MCAs.

Surgical resection was performed for all cases, and chemotherapy and radiotherapy were performed in a few cases [2–29]. Hormone therapy and HER2 targeted therapy have been reported for hormone receptor-positive and HER2 + cases [14, 19, 24]. In the only recurrent case, which was mostly DCIS with only a 5 mm invasive MCA, mastectomy and axillary lymph node dissection were performed without adjunct therapy [21]. Despite the small size of the tumor and the presence of isolated tumor cells in a sentinel lymph node at the time of diagnosis, the tumor recurred 8 years later [21]. Our patient underwent six cycles of chemotherapy and showed no recurrence or metastatic potential thus far. Individualized treatment regimens are still lacking.

Table 1 shows the genomes of only two cases at present. Common genetic variants were *TP53* and *RB1*, which suggests that alterations in tumor suppressor genes, particularly those involved in regulating the cell cycle and chromatin remodeling, are associated with the occurrence of this tumor [18]. In a large NGS project involving more than 10,000 patients with metastatic cancer, *TP53*, *KRAS*, *RB1* and *PIK3CA* were among the top 10 most commonly mutated genes in 62 major solid tumor entities [37]. *TP53* mutations lead to abnormal protein function, and immunohistochemistry showed that p53 protein was overexpressed in our case, which may affect downstream signaling pathways and participate in tumor development [38]. *RB1* mutation may lead to loss of the tumor suppressor function of the RB1 protein, thus promoting excessive cell proliferation, avoiding apoptosis, delaying cell senescence, and participating in the occurrence and development of tumors [39]. Immunohistochemical staining also confirmed RB1 protein loss in our case. Missense mutation in *PIK3CA* (c.3140A > G, p. H1047R), which is located in the phosphatidylinositol 3/4 kinase domain, is a common activation mutation of the *PIK3CA* gene in breast cancer. By enhancing PI3K lipid kinase activity, the PI3K/AKT signaling pathway can be activated to promote the invasion and metastasis of cancer cells and participate in the occurrence and development of tumors [40]. The mutation site (c.35G > T, p.G12V) is the hot spot mutation site of the *KRAS* gene, which has been reported in a variety of tumors, including breast cancer. This mutation can cause impairment of the GP-mediated hydrolytic function of GTP, resulting in increased intracellular RAS-GTP levels, thereby activating the RAS pathway [41]. In addition to mutations in *MAP2K4*, mutations in *KDR*, *PKHD1* and *TERT* may also participate in tumorigenesis. The *MAP2K4* (c.257_258del, p. R86Tfs*7) mutation may reduce the function of MKK4 protein, enhance its mediated cell invasion and participate in the occurrence and development of tumors by promoting the expression of PPARγ [39]. The significance of the other mutated genes is unclear, and they may be related to tumor formation. The gene profile of our case is closer to that of TNBC, especially high-grade TNBC, in which *PIK3CA*, *TP53*, *KRAS* and *RB1* mutations are commonly present [42]. Similarly, this case had a relatively high TMB value of 9.27. According to the immune and gene phenotypes, we further detected PD-L1 (sp142) in this case, and the results showed that the expression rate of PD-L1 (sp142) in immune cells was more than 1%. The tumor genome provided us with other therapeutic clues, such as *PIK3CA* and *KRAS,* which have corresponding targeted inhibitors.

## Conclusion

Mucinous cystadenocarcinoma (MCA) is a rare breast cancer, with only approximately 30 cases reported. Here, we present the gene profile of an MCA case with no recurrence or metastatic tendency after 108 months of follow-up, and a review of the literature helps us better understand the clinical, pathologic, and molecular features of this tumor. A wide panel of immunohistochemical biomarkers should be applied to achieve a correct diagnosis. The genomic characteristics of this tumor are similar to those of common TNBC, in which *PIK3CA*, *TP53*, *KRAS* and *RB1* mutations are commonly present. A better understanding of the genomic characteristics of such tumors could help predict prognosis and guide treatment.

## Data Availability

The dataset supporting the conclusions of this article is included within the article.

## Abbreviations

MCA: Mucinous cystadenocarcinoma
CNB: Core needle biopsy
NGS: Next-generation sequencing
TNBC: Triple-negative breast cancer
EPC: Encapsulated papillary carcinoma
CK7: Cytokeratin 7
CK20: Cytokeratin 20
PET/CT: Positron emission tomography/computed tomography
FFPE: Formalin-fixed and paraffin-embedded
TMB: Tumor mutation burden
